# COMPASS II—Coordination of Medical Professions Aiming at Sustainable Support Protocol for a feasibility study of cooperation between general practitioner practices and community care points

**DOI:** 10.1371/journal.pone.0273212

**Published:** 2022-09-06

**Authors:** Lisa Peter, Judith Stumm, Cornelia Wäscher, Lisa Kümpel, Christoph Heintze, Susanne Döpfmer

**Affiliations:** Institute of General Practice and Family Medicine, Charité - Universitätsmedizin Berlin, Corporate Member of Freie Universität Berlin and Humboldt-Universität zu Berlin, Berlin, Germany; Public Library of Science, UNITED KINGDOM

## Abstract

**Introduction:**

General practitioners (GP) increasingly face the challenge of meeting the complex care needs of multi-morbid patients. Previous studies show that GP practices would like support from other institutions in advising on social aspects of care for multi-morbid patients. Already existing counselling services, like community care points, are not sufficiently known by both GPs and patients. The aim of COMPASS II is to investigate the feasibility of cooperation between GP practices and community care points.

**Methods and analysis:**

During the intervention, GPs send eligible multi-morbid patients with social care needs to a community care point. The community care points report the consultation results back to the GPs. In preparation for the intervention, in a moderated process, GP practices meet with the community care points to agree on information exchange. The primary outcome is the feasibility of the cooperation: Questionnaires will be sent to GPs, medical practice assistances and community care point personnel (focus: practicality, acceptability). Data will be collected on frequency and reasons for GP-initiated consultations at community care points (focus: demand). Qualitative interviews will be conducted with all participating groups (focus: acceptability, satisfaction). The secondary outcome is the assessment of changes in health-related quality of life, social support and satisfaction with care: participating patients complete a questionnaire before and three to six months after their counselling. The results of the study will be incorporated into a manual in which the experiences of the cooperation will be made available to other GP practices and community care points.

**Discussion:**

In COMPASS II, GP practices establish cooperation with community care points. The latter are already existing institutions that provide independent and free advice on social matters. By using an existing institution, the established cooperation and experiences from the study can be used beyond the end of the study.

**Trial registration:**

The trial is registered with DRKS-ID: DRKS00023798, Coordination of Medical Professions Aiming at Sustainable Support II.

## Introduction

It is well known that in Germany, due to demographic change, the proportion of people over 60 is growing in combination with an increase in chronic diseases and multi-morbidity [1–4], while the number of GPs is declining [5, 6]. Providing sufficient and qualified care for these patients is a challenge for the German healthcare system. As early as 2010, Scheidt-Nave called for health research to develop "care models adapted to the needs of specific subgroups of older people," such as patients with multi-morbidity [3].

Results from a prior funding phase of COMPASS showed the GPs need for support in the management of care for patients with multi-morbidity. Interviews with GPs and medical practice assistants in Berlin revealed that the unmet social needs of patients with multi-morbidity are a major concern [7]. GPs do not have sufficient resources to address social issues and would welcome further support, such as better information about and access to social counselling and community services [7–9].

In 2008, German legislation required statutory health and long-term care insurance funds to set up “Pflegestützpunkte” (in this article it is referred to as community care points) in cooperation with the local authorities. The German Social Insurance Code XI defines the following responsibilities of community care points: coordination of home and community-based care, support in nursing and social needs, and support for the utilization of health services [10, 11]. The services of the community care points are independent and free of charge [12]. However, community care points are not well known among the general population [13]. Although community care points offer low threshold community care, they are not used as widely as intended [14]. Focus groups with GPs in Berlin revealed that they have limited or no experience collaborating with community care points [15]. The common practice in dealing with social consulting issues differs between GP practices [7]. Many GPs conduct the consultation on their own, often without an adequately competent background and with restricted time resources [7, 15].

To our knowledge, no project has been undertaken to connect GP practices and community care points with the aim, firstly, to improve utilization of community care points by multi-morbid patients in need and, secondly, to decrease the GPs workload to cope with demographic change.

### Context

COMPASS II is a sub-project of the NAVICARE network for patient-oriented health care research, which aims to reduce barriers and inequalities in the care of patients with age-associated diseases. COMPASS II lays a focus on multi-morbid patients in primary care.

## Methods

### Aim

The aim of COMPASS II is to initiate and explore the feasibility of cooperation between GPs and community care points to meet the social needs of multi-morbid patients. The planned intervention will raise awareness about community care point services among GPs and practice teams and may help to decrease the workload of GPs [16].

### Study design

This is a feasibility study combining descriptive quantitative data and qualitative interviews in a mixed-method approach, addressing the dimensions of acceptability, practicability, satisfaction and demand. Patient outcomes will be investigated by performing two questionnaire-based assessments, at baseline t0 (before counselling at the community care point) and three to six months later (t1).

### Study setting

The study will be conducted in two Berlin city districts, Tempelhof-Schoeneberg and Charlottenburg-Wilmersdorf, comprising 690,931 inhabitants (18.9% of the Berlin population, Status 31/12/2020) and 555 GPs [17–20]. In each district, there are three community care points.

Participating GPs include patients during their usual care consultations. The research team conducts quantitative and qualitative feasibility investigations. The patient assessments will be performed by the medical practice assistants of the participating GP practices.

### Sample size and power considerations

As we plan to conduct an exploratory feasibility study, we did not perform a sample size calculation. Our estimates of the number of participating GPs and patients are pragmatic and are based on the following considerations: from our experience with other studies, we assume, a participation rate of 5–10% related to the 555 GPs listed by the Berlin Association of Statutory Health Insurance Physicians in the study as feasible [19]. We further assume that recruiting 10 patients per GP per 15 months is feasible.

### Recruitment and retention strategies

#### Participants/eligibility criteria

All GPs and community care points in the two Berlin city districts will be invited to participate in the study. There are no exclusion criteria.

Up to 15 patients will be recruited by each GP for the study. The inclusion and exclusion criteria are listed in Table 1.

**Table 1:** Inclusion and exclusion criteria for participants.

**Inclusion criteria**	• at least 18 years old
	• patient in a participating GP practice
	• multi-morbidity (at least two chronic diseases)
	• need for social consultation
	• motivation for a community care point consultation
	• willingness to take part in the study
	• competency to answer the assessment questionnaire at two points in time
**Exclusion criteria**	• living in a nursing home
	• private insurance

Patients who are not able to speak for themselves may be represented by family carers who are spouses/partners or family members living in the same household and who have a legal certificate of representation.

All participants may choose to terminate their participation in the study at any time. Regardless of whether the patients participate in the study or not, they will receive usual care at the GP practice indiscriminately and can seek advice at any community care point.

#### Participant timeline

Community care point enrolment was started and completed in 2020. GP enrolment started in November 2020 and lasted until January 2022. GPs were enrolled into the ongoing intervention in 3 cohorts, starting with the attendance at a cooperation meeting in April, September and December 2021. Patient recruitment started in July 2021 and will end in September 2022. The last patient out will be in December 2022.

#### Recruitment of community care points, GP practices and patients

All community care points (n = 6) in the two city districts were invited to participate in the study. All participating personnel of the community care points received information about the study and were asked to provide written consent.

GP identification was based on the list of all statutory health registered GPs in the two Berlin city districts. GPs were contacted by mail as well as by telephone and were invited to participate in the study. Due to the expected difficulties in recruitment during the Covid-19 pandemic, an adaptive recruitment procedure was applied: recruitment continued beyond the start of the intervention in July 2021 until January 2022 and GPs were invited to participate in the study on multiple occasions. Informative meetings were held for GPs and their medical practice assistants using online formats. At the start of participation in the study, a cooperation meeting between GP practices and the staff of the community care points took place. In the following practice visit, the study nurse provided information about the study and required study documents (study information and consent for patients, templates for documentation). During this visit, she obtained written informed consent from all participating GPs and medical practice assistances. Subsequently, the medical practice assistances were trained in supporting the patient assessment. This training included, among other things, information about when and how to support patients. This could, for example, be to clarify questions of understanding from patients and emphasises the importance of preparing a memo for the second patient assessment (t1).

Patients are identified by the GPs during routine consultations of the patient in the GP practice or during a home visit. If a social counselling need becomes apparent, the patient is asked by the GP to participate in the study. Written consent will be obtained, including a release from confidentiality in the informational exchange with the community care points.

#### Retention strategies

The study nurse conducts both monthly scheduled practice visits and short-term practice visits. The research team provides short-term answers and individual solutions to any upcoming challenges or doubts reported by the GPs or their medical practice assistances. The study nurse is available by telephone and e-mail five days a week for short-term queries.

Financial incentives are provided for participating in the cooperation meetings and the training for medical practice assistances, as well as for each patient inclusion.

### Intervention

#### Cooperation meetings

The intervention started for every participating GP practice by taking part in a cooperation meeting with the community care point personnel and other GP practices participating in the study. The cooperation meeting was facilitated and moderated by the research team. The mode of sending patients, communication, and reports was agreed on between the two parties. Every participating GP and their medical practice assistants were invited to take part in the cooperation meetings to be able to contribute to the agreements. If for any reason a GP was not able to attend any of the meetings, they were asked to comply with the agreements made by other GPs and community care points and got the opportunity of an individual low-threshold meeting with the community care point they prefer to cooperate with. GP practices usually establish cooperation with one to two community care points in their area.

Due to recruiting difficulties during the Covid-19 pandemic, the inclusion of GP practices followed an adaptive approach. Therefore, cooperation meetings were offered consecutively at three points over a period of 9 months (April 2021, September 2021, and December 2021). Intervention then started for the participating GPs after attending the cooperation meeting.

#### Patient visits at the community care points

Eligible patients who give their consent to take part in the study will be asked to make an appointment at the community care point closest to their home or GP practice. GPs will give them a form with a short description of the consultation issues to hand over to the community care point. Alternatively, when required by the patient, an appointment with the community care point will be arranged immediately by the GP by phone. The contact details of the community care point will be handed to the participants by the GP. For all participating patients, the GP practice prepares a memo for the second patient assessment (t1) three to six months after the consultation, which takes place during a routine visit of the patient at the GP practice. Patients who do not give their consent to take part in the study are free to visit a community care point but will not be included in the study.

Consultation by the community care point can be provided at the community care point office (by default) or alternatively by phone or by home visits (depending on the current regulations concerning the Covid-19 pandemic. Consultation may consist of several contacts. The community care point will send a short report about the consultation, the results, measures undertaken, and, where necessary, advice for further proceedings to the GP practice (S1 Fig: Flowchart for recruitment and study process).

### Study structure

COMPASS II is a mixed methods study composed of the following methodological approaches:

#### Quantitative section

*Logbooks on consultations and patient recruitment*. The community care points will compile a logbook of every consultation issue and report consultation frequency during the intervention period without including patient data. In addition, a logbook is kept in each GP practice in which the number of patients considered eligible by the GPs, the number of patients who participate in the study or refuse to participate, and the reasons for refusal and drop-outs (all anonymous) are recorded. Logbooks of GP practices and community care points are used to assess the dimension of demand.

*Questionnaires with GPs*, *medical practice assistants and community care point personnel*. At the end of the intervention, all participating GPs and all medical practice assistants will be asked to fill in a questionnaire on their view of the utilization of the community care points and about their experience of (workload) relief by delegating social consultancy needs to the community care points. The community care point personnel will also receive a questionnaire on their assessment of the use of the community care points within the study. The questionnaires will focus on the dimensions of practicability, acceptability, and satisfaction.

*Patient assessment*. The research team developed a patient assessment to determine the change in the quality of life, satisfaction with care, and social support after the consultation in the community care point. Some of the items were self-constructed. Items from validated questionnaires (Geda-Ehis 2019/2020, Brief Social Support Scale, DeJong Giervield Loneliness Short Scale) were added to the patient assessment [21–23]. One other validated questionnaires was slightly adapted and converted into simpler language to better reach the multi-morbid patients [24].

Patients receive the same questionnaire before and three to six months after their counselling session. The assessment will be collected pseudonymously to allow pre- and post-comparisons. The practices are provided with a list of pseudonyms to be applied to participating patients. The list is held at the practice and the research team has no access to it. The assessments are paper-based and are to be collected during regular visits to the practice by the study nurse.

#### Qualitative section

*Interviews with GP*, *medical practice assistance*, *community care point personnel and patients*. A subgroup of a minimum of eight GPs, a minimum of eight medical practice assistants, and two staff members of each participating community care point (a minimum of 6 staff members), as well as a minimum of 15 patients, have been asked to take part in qualitative interviews on their experience of the cooperation. The first interviews were conducted eight months after the start of the intervention. The last interview will be conducted up to two months after the end of the intervention. The dimensions of acceptability and satisfaction with the cooperation will be assessed during these interviews. Personal data of the respondents have been collected (age, gender, living situation of the patients, professional experience of the GPs and practice assistants, specialization of the GPs, number of staff in the practice, type of practice). All qualitative interviews are semi-structured and an interview guideline is to be used. Interviews have been digitally recorded and transcribed verbatim. All identifying factors have been removed during transcription. Written informed consent for the interviews was already obtained at the study inclusion.

### Outcomes

#### Primary outcome

The primary outcome is the feasibility of the cooperation of general practitioners and community care points in the dimensions of acceptability, demand, satisfaction, and practicability (Table 2).

**Table 2:** Dimensions, indicators of feasibility and methods in COMPASS II.

Dimension	Indicator	Method
Acceptability	• perceived effects and appropriateness of the cooperation and satisfaction with the results, from the view of GPs, medical practice assistances, patients, and community care points	Qualitative interviews
	• % GPs, medical practice assistances, community care point personnel satisfied with the intervention; % GPs being relieved from workload by use of community care point service	Quantitative questionnaires
Demand	• frequency and reasons for consultation of the community care points	Logbooks
Satisfaction	• satisfaction with intervention in the view of GPs, medical practice assistances, patients and community care points	Qualitative interviews
Practicability	• steps taken, communication paths, success or failure of referring patients to the community care points from the view of GPs, medical practice assistances, patients and community care points	Qualitative interviews
		Quantitative questionnaires

#### Secondary outcome

Secondary outcomes are:

- Patient-reported outcomes such as quality of life, satisfaction with care and social support.
- Development of a manual with best practice examples for the cooperation between GPs and community care points planned to be disseminated to other GP practices and the interested public.

### Data analysis plan

The data collected from logbooks will be summarised thematically. Descriptive statistical analyses on recruitment and dropouts will be conducted to determine the feasibility of the study. Data on counselling issues collected from community care points will be grouped and categorized using qualitative methods.

The items of the questionnaire on the feasibility of the cooperation will be analyzed descriptively. Bivariate or multivariate analyses will be conducted exploratively to make assumptions about relationships between feasibility and practice/GP characteristics. IBM SPSS Statistics will be used for the analysis.

The assessment of patient-related outcomes will be analyzed descriptively, comparing the results before and after the patients’ visit to the community care point.

Qualitative interviews will be analyzed using the framework analysis methodology [25]. The analysis takes place within a multidisciplinary team. The researchers familiarize themselves with the audio recordings and transcripts, jointly design coding with an inductive and deductive approach, apply the coding to the material, and bring the analysis to consensus through discussions. For data analyses and coding, the qualitative analysis software program MAXQDA® (VERBI Software GmbH, Germany) will be used.

All analysis steps are discussed in the regular research forum of the Institute of General Practice and Family Medicine of the Charité - Universitaetsmedizin Berlin.

### Data management

Data collection and data entry is performed by the research team and monitored by an experienced researcher from the research team. Each participant (GPs, medical practice assistances, patients, staff members of the community care points and patients) is to be assigned an individual pseudonym. The pseudonym lists remain in the GP´s practices or the community care point offices. The data collected in paper form is stored securely in a locker at the Institute of General Practice and Family Medicine of the Charité - Universitaetsmedizin Berlin or in a password-protected server area. Only the research team has access to the final study dataset.

The coordinator of the NAVICARE network will conduct one independent audit halfway through the study period. The coordinator of NAVICARE is independent of the COMPASS research team and the sponsor.

### Dissemination of results

The relevant results will be published in national and international journals. The manual developed as part of the study with best practice examples for the cooperation between GPs and community care points will be provided to other GPs and community care points in Berlin and Germany.

Additionally, research findings will be disseminated to patients and family caregivers, researchers, healthcare providers and other stakeholders, as well as the general public through the NAVICARE network’s website (https://navicare.berlin/de/), Twitter account (https://twitter.com/NAVICARE_Berlin), annual symposia, presentations at partnering institutions’ events and scientific conferences, and peer-reviewed publications.

### Ethical considerations

Ethical approval was obtained from the Ethics Commission of Charité - Universitaetsmedizin Berlin, Germany (EA1/146/20, July 27, 2020).

## Discussion

In light of demographic change, GPs are facing an increasing workload [5, 26, 27]. To meet these challenges, GPs need and want support [7, 8, 15, 27, 28]. In preliminary studies, there was evidence that, in addition to administrative matters, it is primarily concerning social counselling issues that GPs want to refer to other health care professionals in the care of multi-morbid patients [7, 8, 15]. Zimmermann et al. also showed in a survey of German GPs that social issues are a frequent reason for consultation and that GPs express further interest in institutionalized support for this [9]. In the Dutch program "Welzijn op Recept", GPs can refer patients with psychosocial problems to local social well-being organizations [29]. Here, as in similar projects, a workforce specially trained for the project is needed to assess the individual needs of patients and refer them to the appropriate institutions [29–32]. This requires additional resources, both human and financial [30, 33], which may be available in the context of funded projects but may not prevail beyond the end of the study.

For some years now, there have been community care points in Germany, which are financed by the statutory health insurance funds and local authorities and offer free and independent advice on social issues [10–12]. Although the community points are not very well known thus far [13], in a survey of GPs in Berlin, the majority indicated that community care points could be a source of support for them when caring for patients with complex care needs. [8]. A study in London, which examined the perspective of GPs on barriers to integration between primary and social care revealed poor inter-professional relationships as one barrier [34]. For these reasons, COMPASS II aims at supporting family physicians in establishing cooperation with the already existing institution of community care points. By using an existing institution, the cooperation established during the study can continue beyond the end of the project. The experiences of GPs and community care points gathered in the study will be passed on to other GPs and community care points with the help of a manual. In this way, COMPASS II will continue to contribute to the establishment of further cooperation after the end of the study and will increase the awareness of community care points [16].

One difficulty in the implementation of the study is the Covid-19 pandemic, which is associated with recruitment difficulties of GP practices due to further increased workload [35]. To counter this, the recruitment was adapted, and the GP practices were offered individual solutions as an alternative to the potentially time-consuming cooperation meetings to get in contact with the community care points at the start of the study. In addition, the period during which patients can be enrolled in the study has been extended.

## Supporting information

S1 Fig Flowchart for recruitment and study process. (TIF)

## References

[1] World Health Organization. Multimorbidity [Internet]. Geneva: World Health Organization; 2016 [cited 2022 May 13]. Available from: https://apps.who.int/iris/handle/10665/252275.

[2] Kuhlmey A. [Special care requirements of elderly and old people: as reflected in the new Council of Experts Report]. Z Gerontol Geriatr. 2009;42(6):425–31. German. doi: 10.1007/s00391-009-0072-2 19908080

[3] Scheidt-Nave C, Richter S, Fuchs J, Kuhlmey A. [Challenges to health research for aging populations using the example of “multimorbidity”]. Bundesgesundheitsblatt Gesundheitsforschung Gesundheitsschutz. 2010;53(5):441–450. German. doi: 10.1007/s00103-010-1052-9 20376419

[4] Sass AC, Wurm S, Scheidt-Nave C. [Health and old age: taking stock from the perspective of health reporting]. Bundesgesundheitsblatt Gesundheitsforschung Gesundheitsschutz. 2010;53(5):404–416. German. doi: 10.1007/s00103-010-1049-4 20437022

[5] Beyer M, Erler A, Gerlach FM. [A future concept for primary care in Germany. 1. basics and international models]. Z Allg Med. 2010;86(3):93–8. German. doi: 10.3238/zfa.2010.0093

[6] Advisory Council on the Assessment of Development in the Health Care System. [Coordination and integration—Health care in an ageing society, Special report 2009]. [Internet]. [cited 2022 Jun 28]. Available from: https://www.svr-gesundheit.de/gutachten/sondergutachten-2009/. German.

[7] Stumm J, Thierbach C, Peter L, Schnitzer S, Dini L, Heintze C, et al. Coordination of care for multimorbid patients from the perspective of general practitioners—a qualitative study. BMC Fam Pract. 2019;20(1):160. doi: 10.1186/s12875-019-1048-y 31747886 PMC6865037

[8] Döpfmer S, Trusch B, Stumm J, Peter L, Kuempel L, Grittner U, et al. [Support for General Practitioners in the Care of Patients with Complex Needs: A Questionnaire Survey of General Practitioners in Berlin]. Gesundheitswesen. 2021;83(10):844–853. German. doi: 10.1055/a-1173-9225 32557442 PMC11248836

[9] Zimmermann T, Mews C, Kloppe T, Tetzlaff B, Hadwiger M, von dem Knesebeck O, et al. [Social problems in primary health care—prevalence, responses, course of action, and the need for support from a general practitioners’ point of view]. Z Evid Fortbild Qual Gesundhwes. 2018;131–132:81–9. German. 10.1016/j.zefq.2018.01.008 29510956

[10] Sozialgesetzbuch (SGB XI), Elftes Buch, Soziale Pflegeversicherungen, § 7c Pflegestützpunkte, Verordnungsermächtigung [Internet]. [cited 2022 May 13]. Available from: https://www.sozialgesetzbuch-sgb.de/sgbxi/7c.html.

[11] Braeseke G, Pflug C. Lotsen im Versorgungssystem. Gutachten und Ableitung konkreter Modellprojekte zu Lotsinnen und Lotsen im Versorgungssystem. Gutachten für Senatsverwaltung für Gesundheit, Pflege und Gleichstellung. [Internet]. Berlin: IGES Institut GmbH; 2017 [cited 2022 Jun 28]. Available from: https://www.berlin.de/sen/pflege/_assets/grundlagen/80plus/projektbericht-lotsen-iges.pdf.

[12] Pflegestützpunkte in Berlin: Kompetente Beratung vor Ort, kostenfrei und individuell. [Internet]. [cited 2022 Jun 16]. Available from: https://www.pflegestuetzpunkteberlin.de/ueber-uns/.

[13] Kollak I, Schmidt S. Umfrage zur Bekanntheit von Pflegestützpunkten. Case Manag. 2012;3:132–3.

[14] Kirchen-Peters S, Nock L, Baumeister P, Mickley B. Pflegestützpunkte in Deutschland—Die Sicht der Mitarbeitenden—Der rechtliche Rahmen—Die politische Intention. [Internet]. Friedrich-Ebert-Stiftung WISO-Diskurs; 2016 [cited 2022 Jun 28]. Available from: https://www.iso-institut.de/wp-content/uploads/2016-Kirchen-Peters_Nock_et-al_Die-Sicht-der-Mitarbeitenden.-In_-Pflegestuetzpunkte-Deutschland.-Die-Sicht-der-Mitarbeitenden.-Der-rechtliche-Rahmen.-Die-politische-Intention.pdf.

[15] Stumm J, Peter L, Sonntag U, Kümpel L, Heintze C, Döpfmer S. [Non-medical aspects in the care for multimorbid patients in general practice. What kind of support and cooperation is desired? Focus groups with general practitioners in Berlin]. Z Evid Fortbild Qual Gesundhwes. 2020;158–159:66–73. German. doi: 10.1016/j.zefq.2020.09.001 33187897

[16] Saurman E. Improving access: modifying Penchansky and Thomas’s Theory of Access. J Health Serv Res Policy. 2016;21(1):36–39. doi: 10.1177/1355819615600001 26377728

[17] Statista. Einwohnerzahl in Berlin bis 2020 [Internet]. [cited 2022 May 13]. Available from: https://de.statista.com/statistik/daten/studie/154880/umfrage/entwicklung-der-bevoelkerung-von-berlin-seit-1961/.

[18] Bezirksamt Tempelhof-Schöneberg Berlin. Einwohner*innenzahlen Tempelhof-Schöneberg. Angaben des Amtes für Statistik Berlin-Brandenburg [Internet]. 2021 [cited 2022 May 13]. Available from: https://www.berlin.de/ba-tempelhof-schoeneberg/ueber-den-bezirk/zahlen-und-fakten/einwohner_innen-345126.php.

[19] Kassenaerztlichen Vereinigung Berlin. Arzt- und Psychotherapeutensuche [Internet]. 2020 [cited 2022 May 13]. Available from: https://www.kvberlin.de/fuer-patienten/arzt-und-psychotherapeutensuche.

[20] Bezirksamt Charlottenburg-Wilmersdorf Berlin. Einwohner*innenzahlen Charlottenburg-Wilmersdorf. Angaben des Amtes für Statistik Berlin-Brandenburg [Internet]. 2022 [cited 2022 May 13]. Available from: https://www.berlin.de/ba-charlottenburg-wilmersdorf/ueber-den-bezirk/zahlen-und-fakten/statistiken-fuer-den-bezirk/artikel.1182807.php.

[21] De Jong Gierveld J, Van Tilburg T. The De Jong Gierveld short scales for emotional and social loneliness: tested on data from 7 countries in the UN generations and gender surveys. Eur J Ageing. 2010;7(2):121–130. doi: 10.1007/s10433-010-0144-6 20730083 PMC2921057

[22] Heidemann C, Scheidt-Nave C, Beyer AK, Baumert J, Thamm R, Maier B, et al. Gesundheitliche Lage von Erwachsenen in Deutschland–Ergebnisse zu ausgewählten Indikatoren der Studie GEDA 2019/2020-EHIS. JoHM. Robert Koch-Institut Berlin; 2021 6(3). doi: 10.25646/8456

[23] Beutel ME, Brähler E, Wiltink J, Michal M, Klein EM, Jünger C, et al. Emotional and tangible social support in a German population-based sample: Development and validation of the Brief Social Support Scale (BS6). PLoS One. 2017;12(10):e0186516. doi: 10.1371/journal.pone.0186516 29023540 PMC5638535

[24] Gößwald A, Lange M, Kamtsiuris P, Kurth BM. DEGS: Studie zur Gesundheit Erwachsener in Deutschland: Bundesweite Quer- und Längsschnittstudie im Rahmen des Gesundheitsmonitorings des Robert Koch-Instituts. Bundesgesundheitsblatt—Gesundheitsforschung—Gesundheitsschutz. 2012;55(6–7):775–80. 22736155 10.1007/s00103-012-1498-z

[25] Gale NK, Heath G, Cameron E, Rashid S, Redwood S. Using the framework method for the analysis of qualitative data in multi-disciplinary health research. BMC Med Res Methodol. 2013;13:117. doi: 10.1186/1471-2288-13-117 24047204 PMC3848812

[26] Hobbs FDR, Bankhead C, Mukhtar T, Stevens S, Perera-Salazar R, Holt T et al. Clinical workload in UK primary care: a retrospective analysis of 100 million consultations in England, 2007–14 [published correction appears in Lancet. 2016 Jun 4;387(10035):2292]. Lancet. 2016;387(10035):2323–2330. doi: 10.1016/S0140-6736(16)00620-6 27059888 PMC4899422

[27] Owen K, Hopkins T, Shortland T, Dale J. GP retention in the UK: a worsening crisis. Findings from a cross-sectional survey. BMJ Open. 2019;9(2):e026048. doi: 10.1136/bmjopen-2018-026048 30814114 PMC6398901

[28] Jobst D, Joos S. [Patients’ Social Problems–a Survey in Family Practices]. Z Allg Med. 2014;90(12):496–501. German. doi: 10.3238/zfa.2014.0496–0501

[29] Heijnders ML, Meijs JJ. ’Welzijn op Recept’ (Social Prescribing): a helping hand in re-establishing social contacts—an explorative qualitative study. Prim Health Care Res Dev. 2018;19(3):223–231. doi: 10.1017/S1463423617000809 29268802 PMC6452964

[30] Morse DF, Sandhu S, Mulligan K, Tierney S, Polley M, Giurca BC, et al. Global developments in social prescribing. BMJ Glob Health. 2022;7(5):e008524. doi: 10.1136/bmjgh-2022-008524 35577392 PMC9115027

[31] Roberts T, Lloydwin C, Pontin D, Williams M, Wallace C. The role of social prescribers in wales: a consensus methods study [published online ahead of print, 2021 Apr 15]. Perspect Public Health. 2021;1757913921990072. doi: 10.1177/1757913921990072 33855911 PMC9483681

[32] Hazeldine E, Gowan G, Wigglesworth R, Pollard J, Asthana S, Husk K. Link worker perspectives of early implementation of social prescribing: A ’Researcher-in-Residence’ study. Health Soc Care Community. 2021;29(6):1844–1851. doi: 10.1111/hsc.13295 33528060

[33] MacLeod KE, Chapel JM, McCurdy M, Minaya-Junca J, Wirth D, Onwuanyi A, et al. The implementation cost of a safety-net hospital program addressing social needs in Atlanta. Health Serv Res. 2021;56(3):474–485. doi: 10.1111/1475-6773.13629 33580501 PMC8143691

[34] Naqvi D, Malik A, Al-Zubaidy M, Naqvi Falk, Tahir A, Tarfiee A, et al. The general practice perspective on barriers to integration between primary and social care: a London, United Kingdom-based qualitative interview study. BMJ Open. 2019;9(8):e029702. doi: 10.1136/bmjopen-2019-029702 31434776 PMC6707672

[35] Mughal F, Mallen CD, McKee M. The impact of COVID-19 on primary care in Europe. Lancet Reg Health Eur. 2021;6:100152. doi: 10.1016/j.lanepe.2021.100152 34226894 PMC8245160

