# Supplementary figures and images for: COMPASS II—Coordination of Medical Professions Aiming at Sustainable Support Protocol for a feasibility study of cooperation between general practitioner practices and community care points
Source: PLoS One. 2022 Sep 6;17(9):e0273212. doi: 10.1371/journal.pone.0273212 (PMC9447866; doi:10.1371/journal.pone.0273212)

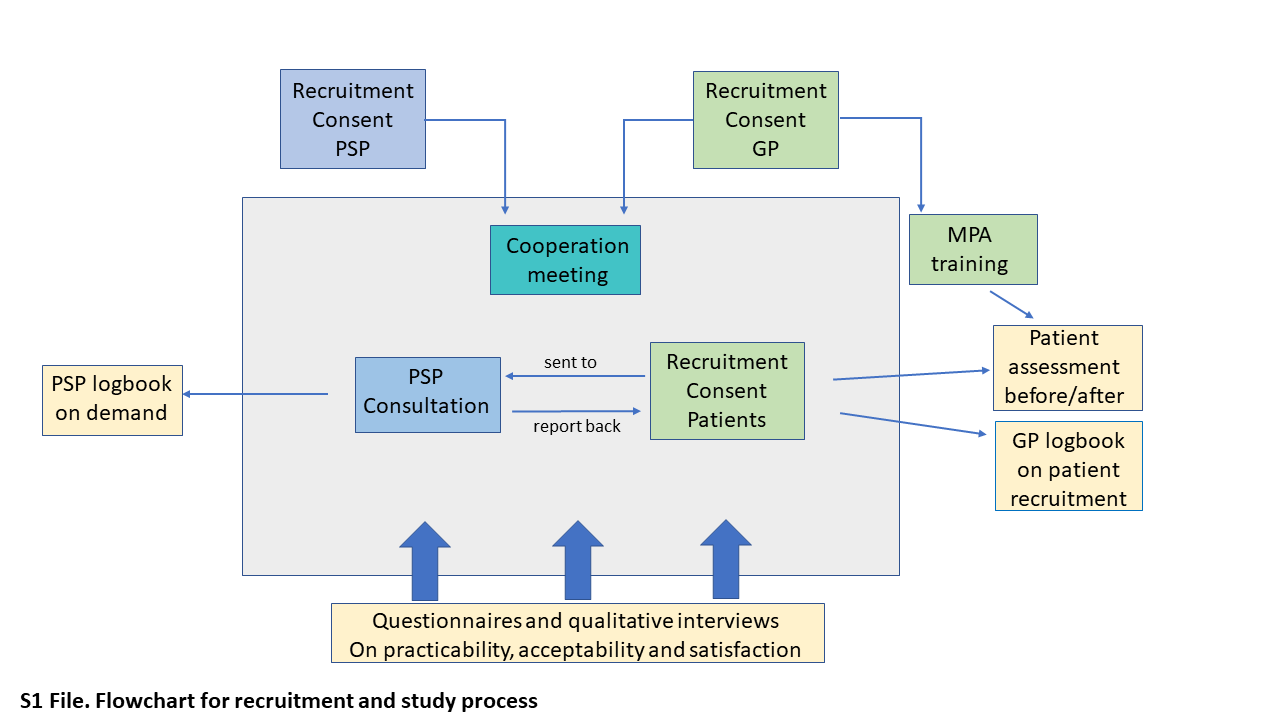

Supplement: S1 Fig — (TIF) [file pone.0273212.s001.tif]
